# The genetic basis for inherited forms of sinoatrial dysfunction and atrioventricular node dysfunction

**DOI:** 10.1007/s10840-015-9998-z

**Published:** 2015-04-12

**Authors:** Raffaella Milanesi, Annalisa Bucchi, Mirko Baruscotti

**Affiliations:** 1 Department of Biosciences, University of Milano, via Celoria 26, 20133 Milan, Italy; 2 Centro Interuniversitario di Medicina Molecolare e Biofisica Applicata, University of Milano, Milan, Italy

**Keywords:** SAN, AVN, Sick sinus syndrome, Atrioventricular block, SAN dysfunction, AVN dysfunction

## Abstract

The sinoatrial node (SAN) and the atrioventricular node (AVN) are the anatomical and functional regions of the heart which play critical roles in the generation and conduction of the electrical impulse. Their functions are ensured by peculiar structural cytological properties and specific collections of ion channels. Impairment of SAN and AVN activity is generally acquired,but in some cases familial inheritance has been established and therefore a genetic cause is involved. In recent years, combined efforts of clinical practice and experimental basic science studies have identified and characterized several causative gene mutations associated with the nodal syndromes. Channelopathies, i.e., diseases associated with defective ion channels, remain the major cause of genetically determined nodal arrhythmias; however, it is becoming increasingly evident that mutations in other classes of regulatory and structural proteins also have profound pathophysiological roles. In this review, we will present some aspects of the genetic identification of the molecular mechanism underlying both SAN and AVN dysfunctions with a particular focus on mutations of the Na, pacemaker (HCN), and Ca channels. Genetic defects in regulatory proteins and calcium-handling proteins will be also considered. In conclusion, the identification of the genetic defects associated with familial nodal dysfunction is an essential step for implementing an appropriate therapeutic treatment.

## Introduction

The sinoatrial node (SAN) and the atrioventricular node (AVN), together with the His–Purkinje fibers, form the cardiac conduction system (CCS). The SAN is the primary rhythm generator of the heart due to the spontaneous electrical activity of its pacemaker cells, while the AVN ensures, after imposing an appropriate frequency-dependent delay, the propagation of the impulse from the atria to the ventricles. At a mature stage, the cells of the CCS, and particularly those of the SAN and AVN, have significant cytological and functional differences when compared to those of the working myocardium: the nodal cells are smaller, have a less-defined sarcomeric organization, and a lower cell-to-cell electrical coupling [1]. Moreover, nodal cells spontaneously generate action potentials due to a specific set of ion channels [2]. Any alteration of the delicate balance between the structural organization and the electrical profile of these cells can thus lead to dysfunctions of impulse generation and conduction. SAN and AVN dysfunctions include a large number of pathological conditions: in most cases they are acquired; but in some cases, they are inherited, and therefore a genetic cause is involved [1, 3–6]. Genetic transmission of a disease is said to be monogenic when the defect is localized in a single gene and to be multigenic when the mutations are present in two or more genes. Despite this simple distinction, there is ample evidence that the phenotypic expression of gene defects is highly complex since incomplete penetrance and high variability are often present. Incomplete penetrance, for example, occurs when an autosomal dominant monogenic disease trait is present in genetically related siblings and only some of them are clinically affected. The presence of variability instead indicates that affected individuals may present a large phenotypic heterogeneity that spans from mild to severe symptoms.

Genetic diseases are commonly thought to have an early onset in life, but this concept does not always hold true since physiological age-dependent molecular and structural remodeling of a given tissue/organ may magnify the pathological impact of a defective gene in the adult or in the elderly. In this regard, it should be considered that the SAN is composed of a relatively low number of cells, and it is known to undergo substantial structural and electrical modifications during ageing, with a reduction in the nodal area and possibly with an increase of fibrotic tissue [1]. Thus, it is conceivable that these structural and electrical age-dependent modifications could in principle set the stage for the late onset of genetic diseases which are not present in young individuals.

The identification of defective genes causing SAN and AVN dysfunctions is starting to emerge from multiple approaches such as genome-wide association studies, the candidate gene approach, and the whole-genome or whole-exome next-generation sequencing. In addition to these genetic studies carried out on humans, a source of valuable information comes from the use of transgenic animal models.

## Anatomy and physiology of the sinoatrial and atrioventricular nodes

**Fig. 1:**
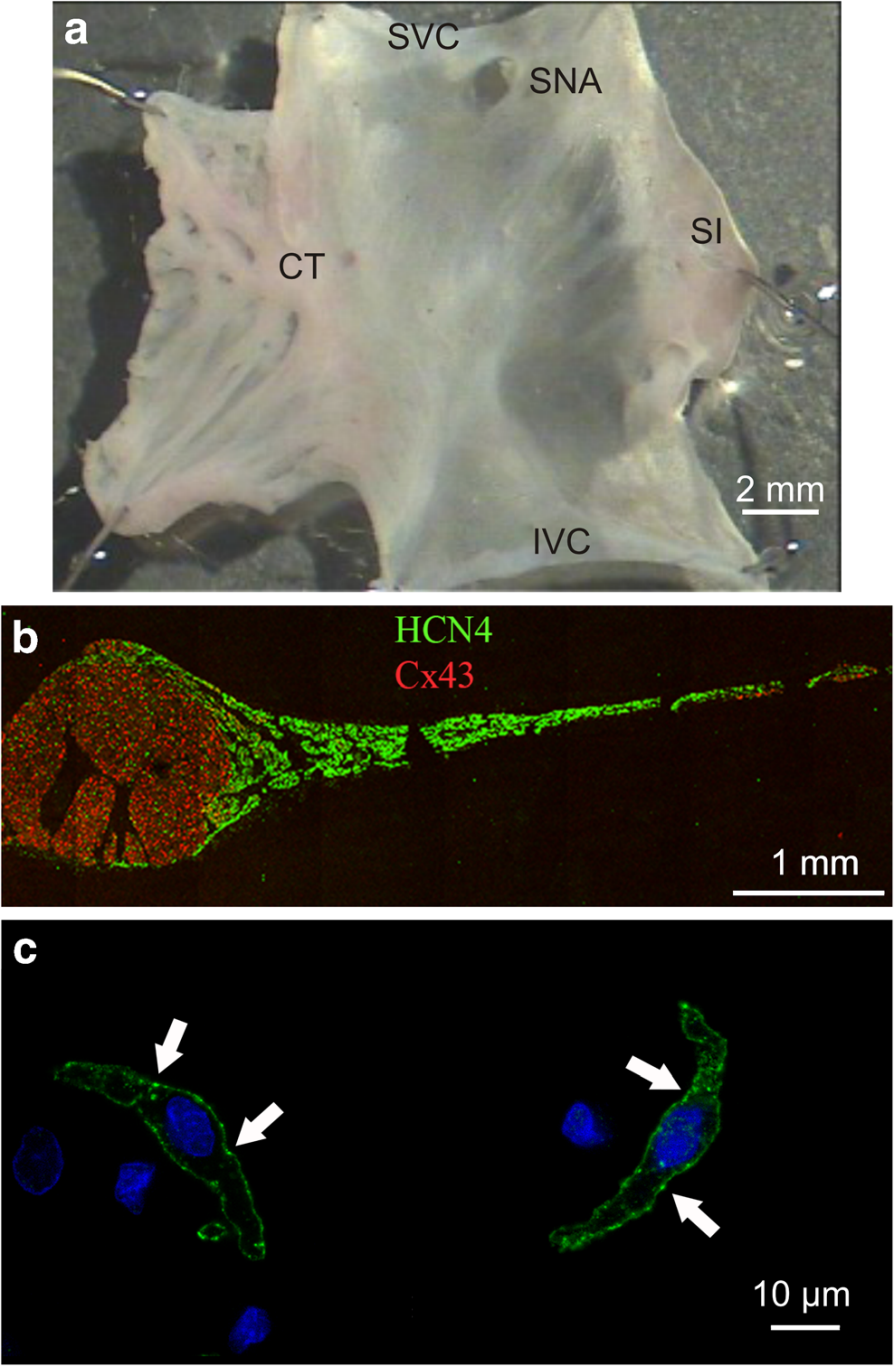
Molecular identification of SAN tissue and cells. **a** Intact SAN isolated from the rabbit heart (endocardial view). *SVC* superior vena cava, *IVC* inferior vena cava, *SI* septum interatrialis, *CT* crista terminalis, *SNA* sinus node artery. **b** HCN4 (*green*) and connexin 43 (*red*) immunolabeling of a SAN tissue slice cut perpendicularly to the intercaval region and extending from the CT to the SI. HCN4 channels are abundantly present throughout the entire SAN area, while connexin 43 is detected exclusively in the CT and in the SI. **c** HCN4 staining (*green*) of rabbit primary pacemaker SAN cells. *Arrows* point to membrane patches with high signal level (hot spots). Nuclei appear in *blue* (modified from [7] with permission)

The SAN is located in the posterior wall of the right atrium, in the intercaval region adjacent to the atrial muscle of the crista terminalis, extending from the superior to near the inferior vena cava, and is organized as a mesh-like structure of sparsely organized myocytes embedded in a dense supporting connective matrix [1, 7] (Fig. 1a). The cellular architecture of the SAN is not uniform since, moving from the center to the periphery, there is a smooth transition from smaller primary pacemaker cells to cells that progressively assume more atrial-like features [1]. Highly specialized central SAN myocytes generate rhythmic pacemaker action potentials that are then orderly conveyed to the rest of the myocardium. It is worth mentioning that when rates are evaluated at the single-cell level, cells from the periphery of the node beat faster than those from the center of the node; however, in *in vivo* conditions, the cells from the periphery have a slower rate due to the hyperpolarizing influence of the surrounding atrial tissue [8].

The main feature of SAN cell autorhythmicity is the presence between consecutive action potentials of a slow diastolic depolarization (pacemaker phase) which sets the cardiac rate. Among the molecular mechanisms that are at work to support this phase, primary roles are played by the pacemaker f-channels (membrane clock) [2, 9] and by intracellular Ca^2+^ release (Ca^2+^-clock) [10]. Molecular studies have shown that specific patterns of mRNA/protein expression can be used as markers of SAN cells. For example, SAN cells are rich in pacemaker “f” (HCN4) channels and Tbx3 factor but lack connexin 43 (Cx43) and atrial natriuretic peptide (ANP) (Fig. 1b, c) [1, 7]. Opposite features are indeed typical of working atrial cells.

**Fig. 2:**
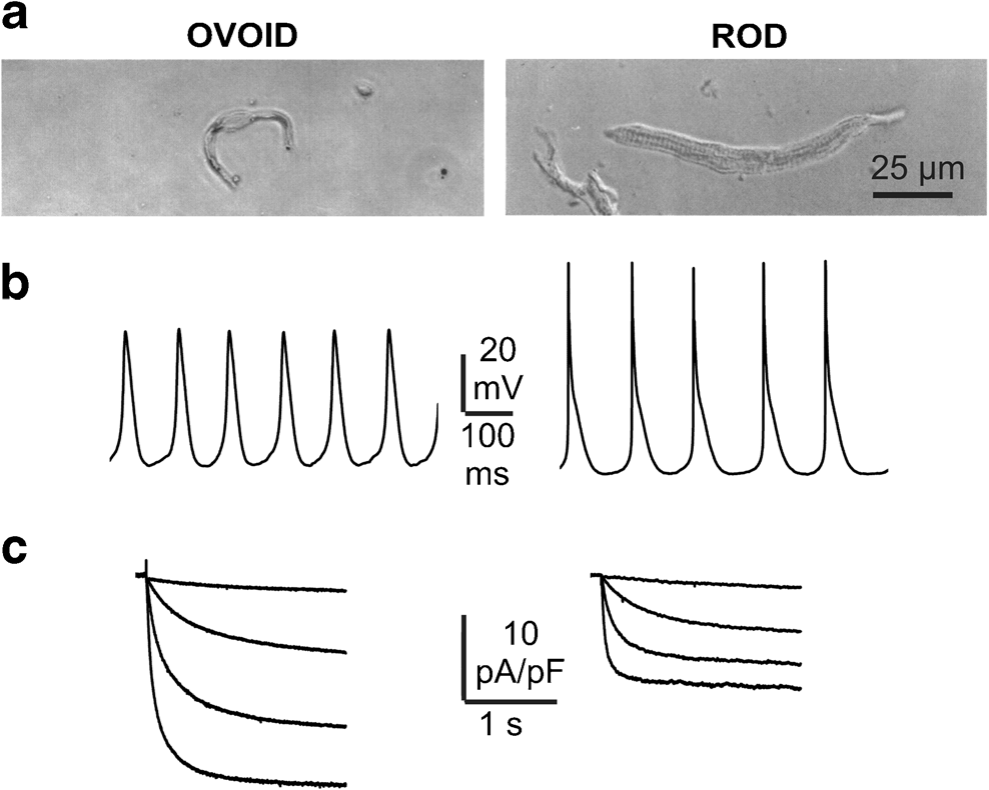
Morphological and electrophysiological characteristics of AVN ovoid and rod cells. **a** Light microscope images of single ovoid and rod cells isolated from the rabbit AVN. **b**, **c** Spontaneous action potentials (**b**) and pacemaker current traces (**c**) recorded from typical ovoid (*left*) and rod (*right*) cells isolated from mouse AVN. Current traces were elicited by hyperpolarizing voltage steps to −65, −85, −105, and −125 mV (holding potential = −35 mV). Ovoid cells beat faster and have larger currents. Recordings were carried out with the whole-cell patch-clamp technique at 35 °C (**a** was from [11] with permission)

The AVN is a complex structure located at the base of the atrial septum at the apex of the triangle of Kock and is the sole anatomical site that allows the electrical continuity (connection) between the atria and the ventricles. The histological organization of the AVN is highly heterogeneous, and this structural complexity (compact node, central fibrous body, penetrating bundle, transitional areas) underlies the existence of two routes for electrical conduction: the fast and the slow pathways [1]. In the presence of pathological atrial/SAN activity, the AVN is endowed with two additional roles: during atrial tachyarrhythmias, it acts as a low-pass filter that protects the ventricles from overwhelming and deadly excitations; during marked sinus bradycardia or arrest, the AVN becomes the pacing unit of the ventricular tissue. Several studies have investigated the different cellular morphologies and action potential shapes of AVN cells. Despite the large heterogeneity, the two most representative cell types of the AVN are those presenting ovoid and rod shapes [11] (Fig. 2a). Both types can be autorhythmic even though the action potential profiles and the underlying pool of ion channels are different. In particular, the action potentials of ovoid cells closely resemble those of sinoatrial cells and thus have a higher pacing rate, while rod cells are more atrial-like and have a slower rate (Fig. 2b). Differences in automaticity are paralleled by the finding that the pacemaker *I*_f_ current is robustly expressed in ovoid cells and less so in rod cells (Fig. 2c).

## Pathology and genetics of SAN and AVN dysfunction

The term SAN dysfunction (or sick sinus syndrome, SSS) is commonly used to identify various pathological conditions related to the inability of the SAN to generate heart rates that are appropriate for the physiologic needs of an individual. Various cardiac disorders such as inappropriate sinus bradycardia and tachycardia, sinus arrest, sinus-exit block, alternating periods of bradycardia and tachycardia, and chronotropic incompetence are recognized as manifestations of sinus dysfunction [12, 13]. Since SSS patients often present with exercise intolerance, presyncope, or syncope, a correct diagnosis represents an important indication for pacemaker implantation. Often, SAN dysfunction is the consequence of structural degeneration or remodeling process due to either pathological conditions such as atrial fibrillation, heart failure, and infarction or the ageing process. In addition, idiopathic degeneration of the SAN with familial inheritance has long been established. Of note is the clinical observation that while congenital idiopathic sinus dysfunction often progresses to atrial standstill, this progression is not typical of the acquired forms [12].

AVN dysfunction is a set of diseases which occur when there is a partial or total block of impulse conduction through the AVN: a condition known as atrioventricular block (AVB). Atrioventricular conduction diseases can be classified on the basis of the degree of block and particularly on the characteristics of the ECG PR interval. AVB most often occurs in association with conduction defects in the His–Purkinje system, a condition that is typical of progressive and non-progressive cardiac conduction disease. In rare cases, a congenital progressive AVB can occur in the absence of concomitant additional conduction defects [14].

Starting from the 1990s, the combined efforts of clinical practice and experimental basic science studies have identified and characterized several causative gene mutations associated with nodal syndromes. These studies have clearly established the concept that one mutation can often cause multiple clinical phenotypes (diseases) and conversely that one disease can often be associated with different types of mutations. This concept thus explains the large overlap and divergence of symptoms that can be observed within one individual, within related siblings, and among unrelated individuals carrying the same mutation/s (genotype).

In the following sections, we will discuss the genetic aspects of the SAN and AVN dysfunction considering both human gene mutations and data from transgenic animal models; information derived from the animal models can indeed be helpful in guiding genetic testing in humans.

## Na^+^ channels

### Cardiac Nav1.5 channels

**Fig. 3:**
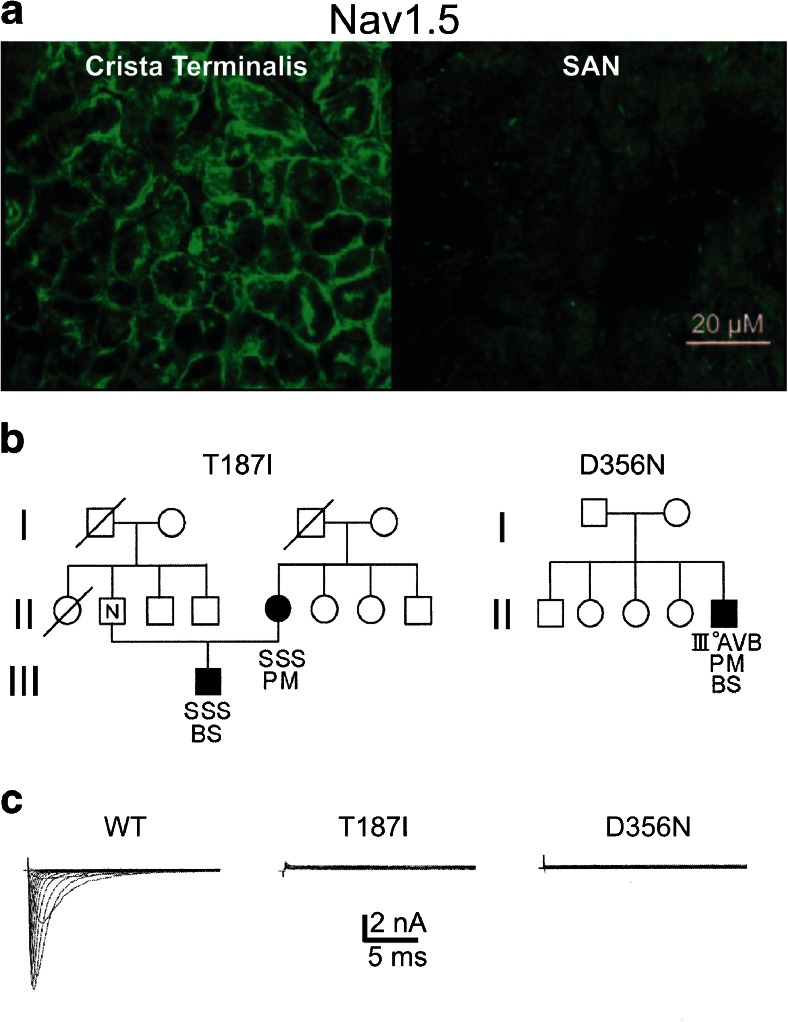
Absence of Nav1.5 channels in human central SAN cells and functional consequences of loss-of-function mutations. **a** Immunolabeling of Nav1.5 channels (*green*) in the human crista terminalis (*left*) and SAN (*right*). Signal detection is strong in the region of the crista terminalis, while in the central SAN area it is virtually absent. **b** Pedigree and clinical features of two families carrying the heterozygous loss-of-functions mutations T187I (*left*) and D356N (*right*) of the Nav1.5 channel. *Circles* female subjects, *square*s male subjects, *diagonal bars* deceased subjects, *solid symbols* mutation carriers, *symbol with “N”* non-mutation carrier, *empty symbols* no DNA test, *AVB* atrioventricular block, *BS* Brugada syndrome, *SSS* sick sinus syndrome, *PM* patient with implanted pacemaker. **c** Whole-cell current samples of wild-type (*WT*) and mutant sodium channels obtained at various membrane potentials (from −90 to +90 mV from a holding potential of −120 mV). Sodium channels were expressed in HEK293 cells in the presence of the auxiliary subunit hβ1. No significant current could be measured in T187I and D356N mutants (**a** was from [19] with permission; **b** and **c** were modified from [20] with permission)

The *SCN5A* gene is located on the short arm of chromosome 3 and encodes the α-subunit of the cardiac sodium channel (Nav1.5). In the working myocardium, Nav1.5 currents ensure the fast depolarization (phase 0) of the action potential, and mutant channels are associated with the Brugada syndrome (BrS) and the LQT3 syndrome [5]. In cardiac SAN cells, the presence of Nav1.5 channels has long been a debated issue. There is now evidence that in most of the animal models investigated, Nav1.5 channels are either absent or scarce in the center of the node, while they are robustly expressed in the periphery where they functionally contribute to impulse conduction [8, 15–18]. In human primary SAN cells, protein and mRNA detection experiments confirmed that Nav1.5 channel signal is small or absent (Fig. 3a) [21]; however, Verkerk *et al*. [22] recorded a Na^+^ current from single human SAN cells and, based on the biophysical features, proposed an Nav1.5 origin [22]. This hypothesis contrasts with the molecular data provided by Chandler *et al.* [21]: a possible explanation for this discrepancy is that the human SAN cells used for patch-clamp experiments [22] were peripheral pacemaker cells. In addition, as will be further discussed in a following section of this review, it is possible that at least part of this current could flow through non-Nav1.5 Na^+^ channel isoforms that are also present in SAN cells.

Despite the lack of Nav1.5 channels in primary pacemaker cells, there is ample evidence that many SCN5A mutations are associated with inherited SAN dysfunction manifestations such as bradycardia and sinus-exit block and that symptomatic and asymptomatic sinus bradycardia is also often observed both in patients affected by Brugada or LQT3 syndrome (Table 1; Fig. 3b, c).

**Table 1:** Mutations of the Nav1.5 channel (SCN5A) associated with nodal and conduction dysfunctions. The functional aspects of all mutations presented in the table have been investigated in heterologous expression studies with the patch-clamp technique. Symptoms listed were identified in the proband and/or in siblings carrying the mutation. Although several mutations displayed biophysical features compatible with both loss of and gain of function; when possible only the major phenotypic impact is listed

Type of mutation, ↑ G-O-F, ↓ L-O-F	Mutation	Impulse generation and conduction dysfunctions	Additional phenotypes	Ref. #
↓	Q55X	Sinus pauses, I° AVB	BrS, intraventricular conduction delay	[23]
↓	R121W	SSS, PCCD	Atrial flutter, VT	[24]
↓	Compound heterozygosity W156X R255W	II° AVB, severe CCD, degenerative changes in the conduction system	VT of unknown origin	[25]
↓	E161K	SB, sinus arrest or exit block, combinations of sinoatrial and atrioventricular conduction disturbances	BrS, paroxysmal atrial tachyarrhythmias	[26]
↓	T187I	SB	BrS	[20]
↓	L212P	Atrial standstill		[27, 28]
↓	T220I	SB, AVB	DCM, AF, BBB	[12, 29]
↓	G298S	Progressive AVB		[30]
↓	D356N	III° AVB	BrS	[20]
↓	R376C	Sinus block, AVB	AF	[31]
↓	R376H	AV-conduction disturbances	BrS, SD	[31, 32]
↑	V411M	II° AVB	LQT3	[33]
↓	T512I H558R	I°, II° AVB	Type 2 conduction system, (Purkinje) block	[34]
↓	G514C	Bradycardia (SB or suppressed conduction through atrial tissues in the vicinity of the sinus node), I° AVB	Slow conduction throughout the atria and ventricles	[35]
↓	R878C	SB, sinus pauses, slow SAN conduction, I° AVB block	Slow intraventricular conduction	[36]
↑,↓	A1180V	AVB	AF, DCM	[37]
↓	D1275N	Sinus node dysfunction, SB, AVB	BBB, AF, atrial flutter, DCM, CHF	[28, 29]
↓	P1298L	I° AVB		[12]
↓	G1408R	I° AVB, CCD	BrS, prolonged QRS	[12, 38]
↓	Compound heterozygosity P1298L G1408R	SSS		[12]
↓	W1421X	SSS, SB, I° AVB, CCD	BBB, SD	[28, 39]
↓	1493delK	SB, I°AVB, CCD	SD	[40]
↑,↓	ΔK1500	Sinus pauses, I° AVB	LQT3, BrS, BBB, SD	[41]
↑	delKPQ1505-1507	PCCD, SB	LQT3, BrS	[42]
↑	delQKP1507-1509	SB, sinus arrhythmia	LQT3	[43]
↓	K1578fs/52	SSS, sinus arrest, I° AVB,	BrS, SD	[20]
↓	D1595N	Progressive AVB		[30]
↓	Compound heterozygosity delF1617 R1632H	Bradycardia, atrial standstill	Prolonged QRS and prolonged His-ventricle conduction time	[12]
↓	R1623X	Sinus arrest, I° AVB	BrS	[12, 20]
↓	Compound heterozygosity R1623X T220I	Absent P waves, sinus pauses		[12]
↓	R1632H	I° AVB		[12]
↓	S1710L	I° AVB	Ventricular fibrillation	[44]
↑	V1763M	Fetal bradycardia, postnatal II° AVB	VT, LQT3	[45]
↑	V1777M	II° AVB	LQT3	[46]
↑	E1784K	SB, sinus pauses	LQT3, SD	[47]
↑	D1790G	Sinus arrest	LQT3	[48]
↑,↓	1795insD	SB, sinus pauses	LQT3, BrS, SD	[49, 50]
↓	L1821fs/10	SB, sinus pauses, CCD, II° AVB	VT, BBB, atrial flutter	[51]
↑,↓	R1860Gfs*12	SB, sinus pauses, I° AVB	AF, atrial flutter	[52]
↓	IVS22_2T_C	AVB, PCCD	BBB	[53, 54]
↓	5280delG	I °AVB, CCD	BBB	[53, 55]

*↑* gain of function (G-O-F), *↓* loss of function (L-O-F), *AVB* atrioventricular block, *BrS* Brugada syndrome, *SSS* sick sinus syndrome, *PCCD* progressive cardiac conduction disease, *CCD* cardiac conduction disease, *VT* ventricular tachycardia, *SB* sinus bradycardia, *DCM* dilated cardiomyopathy, *AF* atrial fibrillation, *BBB* bundle branch block, *SD* sudden death, *LQT3* type 3 long QT syndrome, *CHF* congestive heart failure

Sinus bradycardia could occur as a consequence of either a slower diastolic depolarization or an increased duration of the action potential. *In vitro* characterization of biophysical defects of Na^+^ channels associated with cardiac rate slowing indicates that loss-of-function mutations (typical of Brugada syndrome) are responsible for a slower diastolic depolarization, while gain-of-function mutations (typical of LQT3 syndrome) are responsible for the longer duration of the action potential due to the presence of a non-inactivating sodium current component [49, 56]. In both cases, the overall cycle length increases.

Butters *et al.* [57] have investigated the effect of loss-of-function Nav1.5 channel mutations by developing a mathematical model of the electrical activity of both isolated SAN cells and SAN-atrium two-dimensional preparation. This study has confirmed that at the single cell level only peripheral cells are affected, but in the SAN-atrium simulation the model reproduced a slowing of both SAN rate and impulse conduction leading to SAN exit block or arrest. This study thus provides the rationale that supports the observation that mutations of the hNav1.5 channel are associated with clinical features typical of SAN dysfunction. A thorough investigation of the presence and role of mutant Nav1.5 currents in native human SAN myocytes is obviously difficult; therefore, the use of pacemaker cells derived from induced pluripotent stem cell (IPSc) obtained from patients with SAN dysfunction may represent an important future research tool.

To our knowledge, SCN5A mutations associated with pure sinus tachy-arrhythmias have not yet been identified. Whether this is still a missing piece of data or is an additional indication that Nav1.5 currents are not primarily involved with impulse generation is still an open question.

The functional integrity of Na^+^ channels is also critical for the physiology of the AVN. The first identification of an autosomal dominant SCN5A mutation responsible for AVB was accomplished by investigating the genetic basis of two familial forms of progressive (Lev-Lenegre syndrome) and non-progressive cardiac conduction diseases [53]. After this initial finding, several other mutations have been identified (Table 1). As shown in Table 1, SCN5A mutations associated with AVN dysfunctions have been observed alone or in combination with Brugada and/or LQT3 syndromes. The mechanisms by which loss-of-function and gain-of-function mutations functionally converge toward a common pathological outcome in the AVN (AVB) may be similar to the one described above for SAN bradycardic dysfunction. However, this hypothesis might be an oversimplification given the higher complexity of the physiology of the AVN conduction.

We can thus conclude that the biophysical properties of SCN5A mutant channels associated with nodal dysfunction have been largely identified. However, when the focus is moved from the bench to the bedside, a large overlapping of phenotypes [5], together with incomplete penetrance and variability, often complicate the possibility to adopt a personalized (mutation-tailored) clinical treatment.

### Other Na^+^ channel isoforms and auxiliary β-subunits

The first identification of a non-Nav1.5 isoform was carried out in neonatal rabbit SAN cells, where a Nav1.1 tetrodotoxin (TTX)-sensitive current was shown to be selectively expressed during the first month after birth and to significantly contribute to the pacemaker activity [15, 58, 59]. Several other studies have further investigated the presence of Nav1.1 and of other isoforms in the adult mammalian SAN, and there is now ample evidence that Nav1.1 (SCN1A), Nav1.2 (SCN2A), Nav1.3 (SCN3A), and Nav1.6 (SCN8A) channels are present in SAN cells, although substantial differences in the expression profiles exist among different species [18, 60, 61].

In the human SAN, the presence of detectable albeit low quantity of Nav1.1, Nav1.2, and Nav1.4 (SCN4A) channels was confirmed by Chandler and colleagues [21]. Based on this evidence, it is therefore possible that the INa current originally recorded in the human SAN [22] may be at least partly associated with these isoforms.

The presence of non-Nav1.5 channel isoforms has also been investigated in the AVN of several species, and positive identification has been accomplished for Nav1.1, Nav1.2, Nav1.3, and Nav1.7 (SCN9A) [60, 62]. In humans, Greener *et al.* [63] have confirmed the presence of Nav1.1, Nav1.3, Nav1.6, and Nav1.7, but their quantitative expression is about tenfold lower than the Nav1.5 channels.

Currently, there is no evidence of mutations of non-Nav1.5 isoforms associated with SAN or AVN dysfunction in humans. However, it is interesting to consider that in a mouse model, the conditional and cardiac-specific heterozygous knockout of the SCN1A gene causes a reduction of heart rate, an increase in the PR interval, and an increase in the heart rate variability [64]. An additional confirmation of the pathophysiological roles of Nav1.1 channels comes from the evidence that in a rat model of heart failure (HF), a profound decline of Nav1.1 (−60.7 %) and Nav1.6 (−47.4 %) isoforms likely contributes to the impairment of SAN function [61]. It remains an open question whether a remodeling of Nav1.1 channels also occurs in human HF patients where a depressed SAN function is often observed [65].

We can thus suggest that non-cardiac (non-Nav1.5) Na^+^ channels should also be investigated when looking for the genetic basis of nodal dysfunction.

Na^+^ channel accessory β-subunits are important modulators of the pore-forming α-subunits, and therefore their functional integrity is also extremely relevant in order to ensure proper Na^+^ currents. Loss-of-function mutations of the Na_v_β1 (SCN1B) protein have indeed been reported to be associated with the Brugada syndrome and/or conduction disease [66]. Further indication supporting a pathological role of Na_v_β1 defects comes from a Na_v_β1 null mouse which exhibits both cardiac (prolonged QT and bradycardia) and neurological disorders [67].

## HCN4 channels

**Fig. 4:**
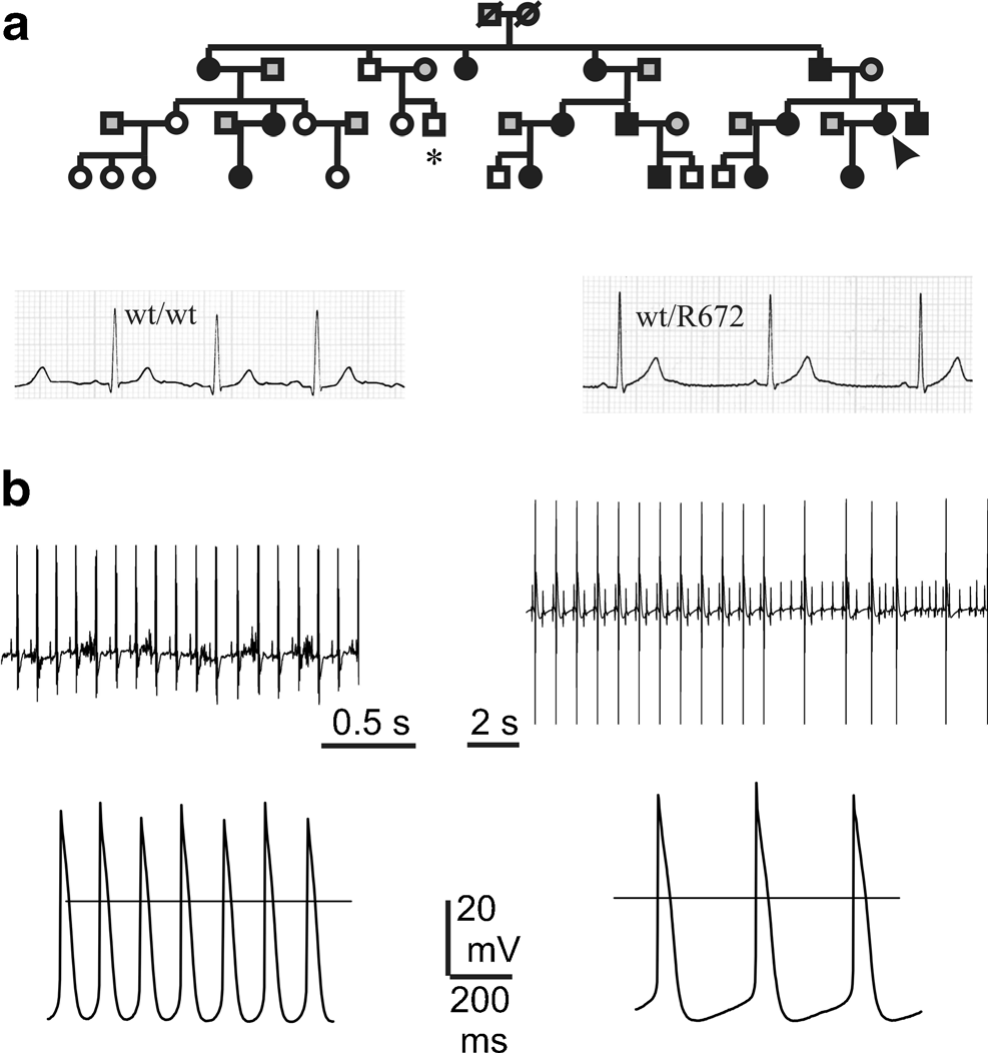
Impairment of HCN4 channel contribution to cardiac pacemaker causes SAN and AVN dysfunction. **a** *Top* pedigree of a four-generation family with an inherited form of sinus bradycardia associated with the S672R mutation of the HCN4 channel. Persons carrying the heterozygous S672R mutation are represented by *solid symbols*, *wt* (control) individuals are indicated by *open symbols*, and *gray symbols* are genetically unrelated individuals. **a** *Bottom* electrocardiograms of a control individual (*asterisk* in the pedigree) and of the proband carrying the mutation (*arrow* in the pedigree); resting heart rates were 80 and 43 beats per minute, respectively. **b** *Top* telemetric ECG recordings from a freely moving transgenic mouse prior to (*left*) and after 4 days of cardiac specific and inducible knockout of the HCN4 channel (*right*). Sinus bradycardia and AVB are evident upon HCN4 knockout. **b** *Bottom* spontaneous action potentials recorded from single SAN cells isolated from a control (*left*) mouse and a transgenic mouse after 4 days of cardiac specific and inducible knockout of the HCN4 channel (*right*). The slowing of spontaneous rate is evident (**a** was modified from [76] with permission; **b** was modified from [78] with permission)

Pacemaker f-channels belong to the hyperpolarization-activated cyclic nucleotide-gated (HCN) channel family and have long been recognized for their primary role in generating and modulating the automaticity of SAN cells [9, 68]. HCN4 is the principal isoform (~80 % of the total *I*_f_ current) expressed in the conductive tissue and in particular in the SAN, and screening analysis performed on arrhythmic patients has identified several HCN4 mutations associated with symptomatic and non-symptomatic alterations of the sinus rhythm (Table 2; Fig. 4a).

**Table 2:** Mutations of the pacemaker (HCN4) channels associated with nodal dysfunctions. The functional aspects of all mutations presented in the table have been investigated in heterologous expression studies with the patch-clamp technique. Symptoms listed were identified in the proband and/or in siblings carrying the mutation

Type of mutation, ↑ G-O-F, ↓ L-O-F	Mutation	Impulse generation and conduction dysfunctions	Additional phenotypes	Ref. #
↓	A414G	SB	LVNC, AF, atrial standstill	[69]
↓	G480R	SB		[70]
↓	Y481H	SB	LVNC, AF, degeneration of the mitral valve; polymorphic ventricular, extrasystoles during exercise	[69]
↓	G482R	SB, I°AVB, impaired chronotropic capacity	LVNC, intermittent ectopic atrial rhythms, mitral valve prolapse, out-of-hospital cardiac arrest	[69, 71]
↓	A485V	SB	Out-of-hospital cardiac arrest during extreme exercise, inducible AF, paroxysmal AF	[72]
↓	K530N	SB, sinus pauses, tachycardia-bradycardia syndrome	Persistent AF	[73]
↓	D553N	SB	QT prolongation; torsade de pointes, cardiac arrest for 40 s followed by polymorphic VT	[74]
↓	573X	SB, chronotropic incompetence during exercise	Intermittent AF	[75]
↓	S672R	SB		[76]
↓	695X	SB, episodes of distinctive sinus arrhythmia linked to adrenergic stress	LVNC, susceptibility to atrial and ventricular premature beats, mitral valve prolapse	[71, 77]

*SB* sinus bradycardia, *LVNC* left ventricular noncompaction cardiomyopathy, *AF* atrial fibrillation, *AVB* atrioventricular block, *VT* ventricular tachycardia

All the mutations identified thus far are of the loss-of-function type, and their resulting phenotypes are compatible with a reduced contribution of the pacemaker current to the diastolic depolarization of SAN cells in resting conditions.

Recently, it has been demonstrated that selective knockout of HCN4 channels in adult transgenic mouse causes sinus bradycardia and AVB that progresses from PQ prolongation to complete heart block (Fig. 4b) [78]. While the sinus bradycardia was obviously predictable, the presence of the AVB was completely unexpected. The evidence that a reduction of the pacemaker current (of about 75 % both in SAN and AVN cells) interferes with the conduction of the impulse through the AVN is not of immediate interpretation. Indeed these data challenge the current view that the sole role of the HCN4 current is to ensure subsidiary pacemaker AVN activity when the primary impulse from the SAN is pathologically impaired. Although it is only speculative, a possible interpretation of the knockout data is that proper SAN impulse propagation throughout the AVN requires AVN cells to be partially depolarized by their own spontaneous diastolic phase; if this spontaneous background depolarization is altered, then also impulse propagation through the AVN is impaired.

Of extreme clinical interest is the association of atrial fibrillation (AF) with the presence of spontaneous arrhythmogenic activity localized in proximal part of the pulmonary veins (PVs) near the left atrium. The identification of this region as an important therapeutic target for the treatment of AF has guided the seminal work of several groups which have demonstrated that focal radioablation of the tissue around the PVs often successfully resolves AF. Investigation of the cytological organization of the pulmonary sleeves in tissue samples from human AF patients has confirmed the presence of cells similar to those of the conduction tissue [79]. This information, together with the established evidence that in small mammals the *I*_f_ current is highly expressed in this region, raises the question whether familial forms of AF arising from the pulmonary vein sleeves are related to a pathological alteration of the *I*_f_ expression [80].

## Ca^2+^ channels and Ca^2+^ handling proteins

### Ca^2+^ channels

Studies on animal models have shown that voltage-dependent L- and T-type Ca^2+^ currents and intracellular Ca^2+^ oscillation provide an important contribution to support the pacemaker activity of sinoatrial node cells. In central nodal cells, Ca^2+^ currents (L-type: Cav1.2 and Cav1.3 channels; T-type: Cav3.1 channels) indeed provide a depolarizing force that both supports the second part of the diastolic depolarization and ensures the upstroke of the action potential [81, 82].

Mangoni *et al.* have shown that transgenic Cav3.1^−/−^ mice, lacking the T-type calcium currents, present with a moderate reduction of the intrinsic heart rate and have a first-degree AVB [83]. No evidence of mutations affecting Cav3.1 channels has been reported in humans. However, a possible pathological association between loss-of-function of T-type channels and bradycardia and atrioventricular block is found in pediatric patients affected by congenital heart block [84] caused by IgG-induced inhibition of calcium T- and L-type channels in children of mothers affected by an autoimmune disease of rheumatic origin [85].

**Fig. 5:**
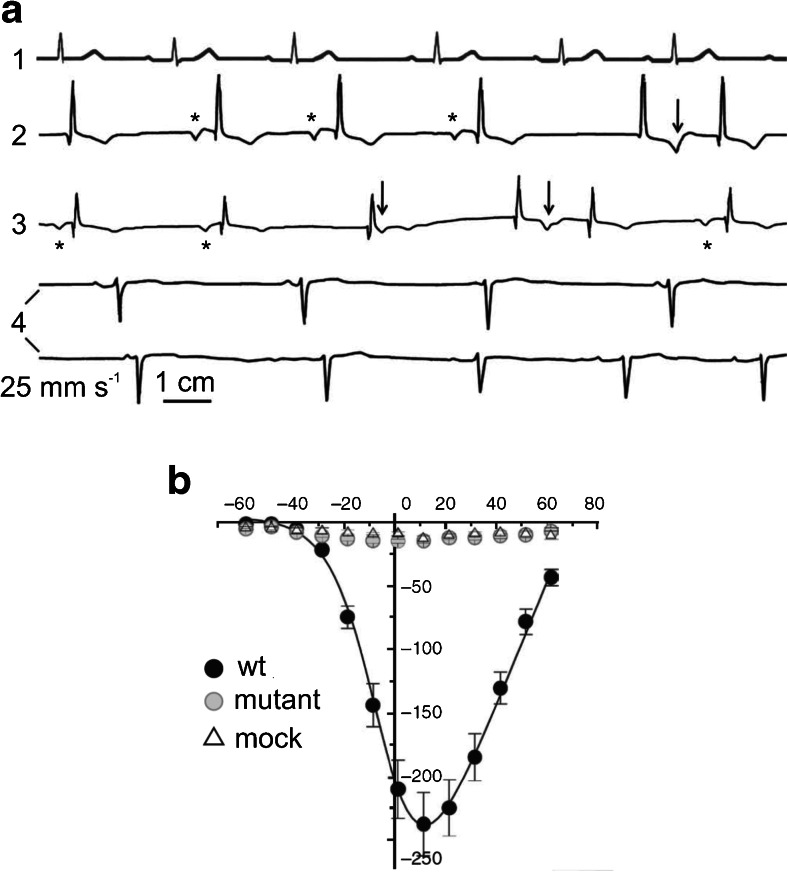
Clinical and cellular phenotypic expressions of mutant Cav1.3 channels associated with SAN dysfunction. **a** Electrocardiograms obtained from a wild-type subject (*1*) and three homozygous carriers (*2*, *3*, *4*) of the c.1208_1209insGGG (p.403_404insGly) mutation of the CACNA1D gene (Cav1.3 calcium channel). Mutant carriers are clinically affected by SAN dysfunction. *Asterisks* identify P waves that precedes ventricular activation (QRS complexes), and *arrows* identify overlapping of P and T waves. A large variability of the P–P and R–R intervals are observed in arrhythmic patients. **b** Current–voltage relation of heterologous expression of Cav1.3 channels; no significant current can be measured in mutant p.403_404insGly channels (modified from [86] with permission)

In 2011, the first homozygous loss-of-function mutation (1208_1209insGGG) of the human L-type Cav1.3 channel was identified in patients affected by the following symptoms: severe to profound deafness, resting bradycardia (32–52 bpm), increased heart rate variability, altered atrioventricular conduction, and junctional escape rhythm; SAN arrest and exit block were also observed [86] (Fig. 5). This complex disorder was termed SAN dysfunction and deafness syndrome, and the presence of cardiac as well as hearing impairment is reminiscent of the phenotype observed in Cav1.3 knockout mice which also show SAN and AVN dysfunction as well as deafness [87, 88].

### Ca^2+^ handling proteins

In working myocytes, the intracellular control of the Ca^2+^ concentration is a fundamental aspect of the excitation–contraction coupling process. Intracellular Ca^2+^ oscillations also represent an important mechanism that, together with plasma membrane ion currents, governs the automaticity of spontaneously beating SAN cells. It is therefore expected that mutations of Ca^2+^ handling proteins may have important pathological consequences both in working myocytes and in myocytes of the conduction tissue.

Calsequestrin (CASQ2) is a sarcoplasmic Ca^2+^-binding protein, and ryanodine receptor type 2 (RYR2) is a calcium channel of the sarcoplasmic membrane. Loss-of-function mutations of CASQ2 and RyR2 have been associated with catecholaminergic polymorphic ventricular tachycardia (CPVT), and CPVT patients often present with SAN bradycardia [89, 90].

The Na^+^/Ca^2+^ exchanger (NCX1) is an important effector of the Ca^2+^-clock mechanism since it provides a depolarizing current that contributes to the pacemaker activity of SAN cells [10]. At present, there are no reports on mutations of NCX1 associated with nodal dysfunction; however, recent studies in mice have shown that partial ablation (70–80 % knockout) of NCX1 does not affect both *in vivo* and *ex vivo* basal heart rates but severely impairs the β-adrenergic-induced response, while its complete knockout fully eliminates the SAN pacemaker activity [91, 92]. Given the substantial difference between the two phenotypes, it can be hypothesized that in the full knockout model, the severe impairment of SAN cell function can be associated both to the lack of electrogenic activity of the NCX1 pump and to substantial impairment of multiple additional pathways associated with the altered Ca^2+^ homeostasis.

## Popdc proteins and twin-pore potassium (K2P) channels

The Popeye domain-containing gene (Popdc) family is a class of membrane proteins largely present in the heart with an evolutionary conserved structural element (the Popeye domain), which binds to the cellular second messenger cAMP. Interestingly, there is a structural similarity between the Popeye domain and the cyclic nucleotide binding domains of the cardiac pacemaker (HCN) channels and of the cAMP-dependent protein kinase (PKA) [93]. In mice, Popdc1 and Popdc2 isoforms are highly expressed in the entire cardiac conduction system, and their knockout induces the appearance of severe sinus node dysfunction, but only in the presence of stress-induced conditions and in an age-dependent manner [94]. The histological analysis of these arrhythmic mutant mice demonstrates a loss of HCN4 positive pacemaker cells [94]. Also, functional knockout of Popdc2 in the zebrafish model also induces severe baseline brady-arrhythmias and AVB [95].

One hypothesis that can explain how the Popdc pathway modulates the cardiac automaticity is based on the observation that Popdc proteins are functional modulators of TREK-1 channels which are members of the twin-pore potassium ion (K2P) channel family [94], and K2P channels are known to control the excitability, the resting membrane potential, and the repolarization phase of cardiomyocytes [96]. Although TREK1 channels appear to be absent from the human SAN, other members of this family such as TWIK1 and TASK1 have been identified [21]. Interestingly, studies of SAN remodelling associated with heart failure (HF) in a rat model have shown that TWIK1, TWIK2, and TASK1 are upregulated during HF and could thus represent a mechanism contributing to the decreased intrinsic heart rate associated with HF [97]. Also indicative is the finding that in *Drosophila* a K2P channel (ORK1) directly contributes to setting the duration of the diastolic depolarization [98]. Additional proposed mechanisms of the action of Popdc proteins include (a) possible interactions with other ion channels such as HCN or Na channels, (b) a cAMP-dependent role as transcriptional regulators since Popdc proteins have been found also within cell nucleus, and (c) interaction with caveolin-3 since loss of Popdc1 impairs both the caveolar size and the correct trafficking of proteins normally associated with caveolae [99, 100].

Whether these genes are also functionally relevant in the cardiac settings of human nodal diseases remains to be determined.

## Lamin, emerin, nesprin

Lamin A/C, emerin, and nesprin proteins are part of an intricate protein network of muscle cells that connect the nuclear envelope to the cell nucleo- and cytoskeleton. Interestingly, lamins are considered to be the ancestors of all intermediate filaments. Mutations affecting the correct functions of these genes have been linked to skeletal muscle (Emery–Dreifuss muscular dystrophy) and cardiac diseases including conduction dysfunction [6]. A clear understanding of the functional role and pathological aspects of these proteins is still lacking; however, it may be that the integrity of this protein network is necessary for proper tissue-dependent chromatin dynamics and gene expression [101].

## Ankyrin-B and caveolin-3

Ankyrins are a class of proteins ubiquitously expressed in excitable and non-excitable cells whose function is to connect integral membrane proteins to the cytoskeleton. Far from being purely passive structural elements, ankyrins contribute to maintaining the proper spatial and functional organization of ion channels, transporters (Na^+^/K^+^ exchanger, Na^+^/K^+^ ATPase), and Ca^2+^ handling proteins of the sarcoplasmic reticulum such as inositol 1,4,5, trisphosphate (InsP3) receptor, and ryanodine receptor (RyR2) [102]. It is thus not surprising that loss-of-function mutations of ankyrin-B, an isoform largely expressed in the heart, profoundly alter the proper homeostatic control of Ca^2+^ and Na^+^ ions, thus causing a critical substrate for the occurrence of mechano-electrical instability. This condition can then precipitate to a pathological manifestation known as Ankyrin-B syndrome. Symptoms associated with this syndrome include sinus arrhythmias, prolonged QT, torsade de pointes, and atrial fibrillation [102, 103]. Sinus node dysfunction is observed in a relevant fraction (up to 75 %) of individuals affected by the Ankyrin-B syndrome, while QT prolongation is rarer and only occurs in the most severe clinical manifestations [103–105]. Of relevance is also the evidence that a distinct ankyrin protein (ANK-G) selectively interacts with Nav1.5 channels (and with Nav.12, Nav1.6), and patients presenting a mutation in the Nav1.5 channel that disrupt this interaction present with Brugada syndrome [102].

Caveolin-3 (CAV3) are integral membrane proteins abundantly present in cardiac and skeletal myocytes where they contribute to the formation of caveolae which are Ω-shaped invaginations of the membrane. Caveolar organization of the membrane favors the co-localization of ion channels, transporters, receptors, and several other proteins in order to ensure optimal spatial and time confinement of local signal transduction. In the heart, CAV3 proteins are virtually present in all myocytes and particularly in SAN cells [106]. Genetic defects of caveolin-3 have been associated both with LQT9 and with sudden infant death syndromes, and the underlying mechanism responsible is the large increase of the late component (up to fivefold) of the Nav1.5 sodium current which is caused by these mutations [107, 108]. Interestingly, patients carrying the T78M mutations also displayed marked sinus bradycardia. The proposed mechanism for this T78M-dependent bradycardia is a modification of the Nav1.5 current which is expressed in peripheral SAN cells. Since Cav3 is also largely present in the center of the SAN, and it functionally interacts with several other ion channels, among which the HCN channels [109], it is also possible that the sinus bradycardia observed in patients carrying the T78M mutation might be caused by the disruption of these additional interactions with other channels.

## Protein kinase PRKAG2

The 5′ AMP-activated protein kinase (AMPK) is a metabolic sensor that couples cardiac cell physiology to the metabolic state of the heart and is activated by AMP and inhibited by ATP. If the energetic content of the cells becomes too low, the AMPK will counteract this condition by limiting ATP consumption and by activating ATP-producing pathways. Mutations of the PRKAG2 protein are often associated with ventricular pre-excitation (typical of the Wolff–Parkinson–White syndrome) and with AVB and sinus bradycardia [3, 6, 110]. Cardiac hypertrophy of working and conductive myocytes due to an exaggerated glycogen deposition is also typically observed. At present, a convincing molecular explanation for the clinical phenotypes is still missing. While it is possible that the structural modifications due to the hypertrophic state can lead to electrical remodeling and thus to conduction disorders, it cannot be excluded that mutant PRKAG2 proteins may play a role in controlling the expression and modulation of ion channel of conductive cardiomyocytes.

## Conclusions

The SAN and the AVN together with the His–Purkinje system ensure the appropriate generation and delivery of the electrical impulse to the working myocardium. The physiological functions of these strategic regions can occur only if all the structural and electrical features of these cells are not impaired by acquired or inherited dysfunctions. In recent years, combined efforts of clinical practice and experimental basic science studies have identified and characterized several causative gene mutations associated with the nodal syndromes. One concept that emerges strongly from these studies is that even a single mutation can induce several phenotypical manifestations, and therefore overlapping syndromes are often simultaneously present. The presence of incomplete penetrance and variability often further increases this complexity. Taken together, these concepts explain the clinical observation that “pure nodal dysfunctions”, i.e., dysfunctions of the SAN and AVN in the absence of any additional cardiac disorders, are rare. Despite this complexity, the genetic identification of mutations associated with inherited nodal dysfunctions is becoming a valuable clinical element that may be utilized in order to optimize the clinical therapeutic approach for each patient.
